# A rare case of ectopic pregnancy in a caesarean section scar: a case report

**DOI:** 10.1259/bjrcr.20170010

**Published:** 2017-08-03

**Authors:** Omer A. Mahmoud, Mustafa Z. Mahmoud

**Affiliations:** ^1^Medical Ultrasound Imaging Department, Dr. Mohamed Abdel Mageed Ali Medical Complex, Alnohood, Sudan; ^2^Radiology and Medical Imaging Department, College of Applied Medical Sciences, Prince Sattam bin Abdulaziz University, Al-Kharj, Saudi Arabia

## Abstract

Intramural pregnancy with implantation in a previous caesarean section scar is probably the rarest location for an ectopic pregnancy. Little is known about its incidence and natural history. This rare condition may be accompanied by serious clinical events, such as rupture of the uterus and unrestrainable haemorrhage, which are sometimes treated with hysterectomy causing sterility. Most treatment choices are effectual, such as dilatation and curettage (D and C), excision of trophoblastic tissues using either laparotomy or laparoscopy, systemically administered methotrexate and, more recently, uterine artery embolization. Here, we report a case of an ectopic pregnancy that occurred in the scar of a previous caesarean section, which was diagnosed by transvaginal sonography.

## Case presentation

A 33-year-old Sudanese woman, gravida 8, para 7 with 2 months of amenorrhea, was admitted to hospital following complaints of minimal vaginal bleeding, lower abdominal pain, nausea and vomiting on the day of admission. She had undergone a caesarean section 3 years prior. The patient’s physical examination revealed abdominal distention and generalized tenderness during palpation. A bimanual vaginal examination demonstrated an enlarged uterus that seemed to be consistent in size with 8 weeks’ gestation. The patient’s serum beta human chorionic gonadotrophin (β-hCG) level was 7,928 mIU ml^–1^.

## Investigations

**Figure 1.**
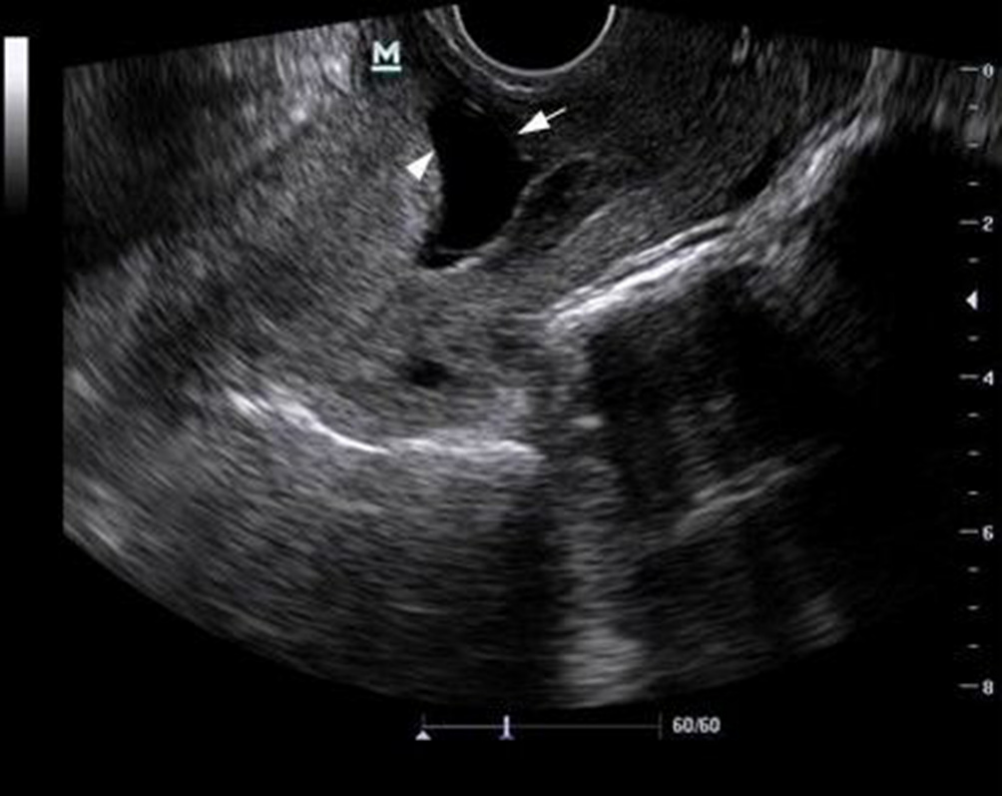
Transvaginal sonography showing an empty uterine cavity with a gestational sac located in the anterior myometrium of the lower uterine segment (solid arrow). The examination also demonstrates a thin anterior myometrium (arrowhead) with no fluid collection in the posterior cul-de-sac.

The patient was examined using transvaginal sonography (TVS), which revealed an empty uterine cavity and a 28 mm, well-defined gestational sac located in the anterior myometrium of the lower uterine segment (Figure 1).

**Figure 2.**
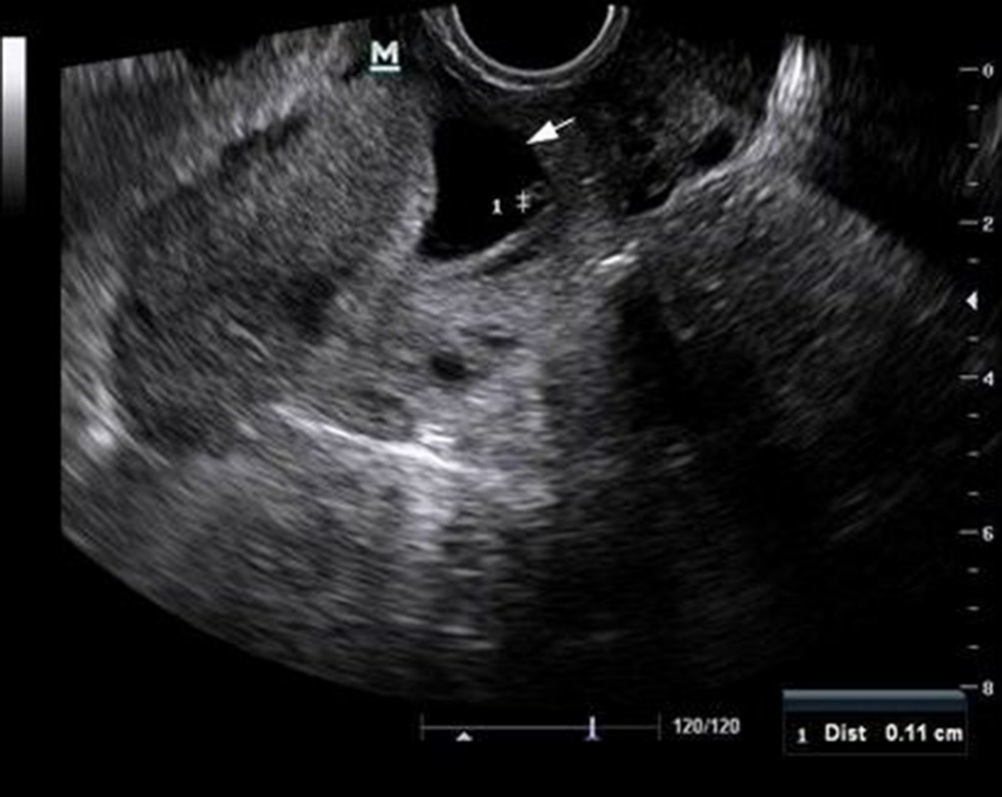
Transvaginal sonography showing an ectopic gestational sac (solid arrow) with an embryo pole (crown-rump length of 1.1 mm) and a yolk sac.

The gestational sac contained an embryo (average gestational age of 4 weeks, 3 days) with positive cardiac activity, a yolk sac and a crown-rump length (CRL) of 1.1 mm (Figure 2).

**Figure 3.**
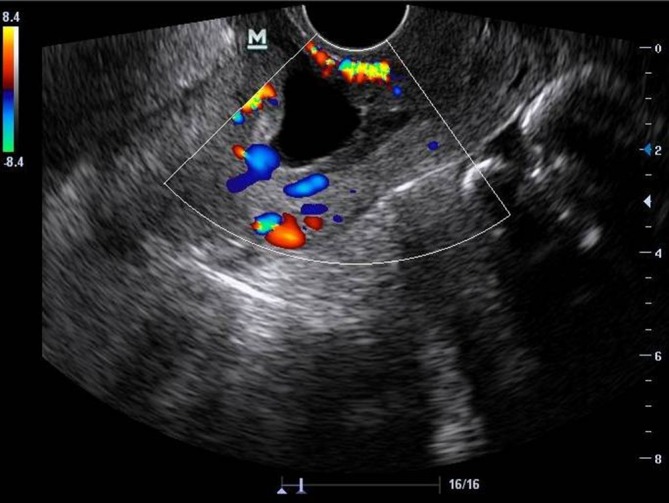
Colour Doppler imaging showing a rim of vascularity, representing a choriodecidual reaction compatible with ectopic pregnancy.

A thinned myometrium (0.9 mm) was detected anterior to the gestational sac during the ultrasound scan. No abnormal fluid collections were detected in the posterior pouch of Douglas (Figure 1). Colour Doppler imaging demonstrated a rim of vascularity, which was indicative of choriodecidual reaction, and there was also increased blood flow with hypervascularization around the gestational sac (Figure 3). These vascular characteristics, which represent the peculiarity of the described TVS findings, were fundamental for considering a diagnosis of ectopic pregnancy in a caesarean scar.

## Differential diagnosis

An ectopic pregnancy in a caesarean section scar is typically the result of a particular complication associated with pregnancy following caesarean delivery, as the gestational sac is implanted in the hysterotomy scar, known as a caesarean scar pregnancy (CSP). The clinical presentation of an ectopic pregnancy in a caesarean section scar ranges from vaginal bleeding with or without pain to uterine rupture and hypovolemic shock. The absence of a particular clinical feature indicative of CSP highlights the importance of TVS and Doppler imaging in establishing a correct diagnosis.^1^ Most of the cases of CSP that have been reported were diagnosed early in the first trimester.^2^ A diagnosis of CSP is made by sonographically visualizing an enlarged hysterotomy scar with an embedded mass. The main differential diagnosis includes cervical ectopic pregnancy and placenta accreta. The diagnostic hypothesis may be confirmed by MRI or during laparoscopy and/or laparotomy.^3^

## Treatment, outcomes, and follow-up

Following the detection of an ectopic pregnancy in a caesarean section scar, the possibility of a ruptured scar ectopic pregnancy was maintained, and an exploratory laparotomy conducted via a transverse incision was performed. Intraoperatively, a uterine scar—including a gestational sac with a living embryo—was confirmed. The uterus was evacuated and the uterine defect was repaired in two layers. After the intervention, the patient was hospitalized for 3 days and treated with antibiotics and heparin, with subsequent discharge in good health with no complications. Follow-up with ultrasonography and colour Doppler of the pelvis showed no complications. Our patient was advised abstinence until resolution of the CSP.

## Discussion

The rate of CSP is expected to rise in the future due to the increasing number of caesarean sections; however, the ideal treatment protocol for this type of pregnancy is still unknown. The underlying reasons for the ectopic implantation of the conceptus into the caesarean scar, as well as its diagnosis and treatment, need further evaluation. Indeed, the exact cause of CSP is ambiguous. It was hypothesized that a microscopic defect of the microtubular tract in the caesarean scar might be related to CSP, as there is a leading invasion of the conceptus into the myometrium.^4^ However, the most widely accepted theory seems to be that the blastocyst invades the myometrium through a microscopic dehiscent tract, which may have resulted from the trauma of a previous caesarean section or any other uterine surgery, or it may even result following manual removal of the placenta.^5,6^

A rapid and accurate diagnosis of CSP may prevent serious clinical complications, enabling the establishment of more conservative therapy. The typical manifestation of CSP is severe vaginal haemorrhage. Accurate diagnosis of CSP is initiated by applying TVS with Doppler.^7^ The TVS-based diagnostic criteria used to differentiate CSP from an ectopic pregnancy implanted in the uterine endocervix includes an empty uterus with a gestational sac situated anterior to the uterine isthmus, and it presents with peripheral hypervascularity on Doppler (the “ring of fire” sign). Laparoscopy or laparotomy may be performed if TVS or MRI failed to identify CSP.^8,9^ Due to the dangers of uterine rupture and unrestrainable haemorrhage, the treatment modalities for CSP are either medical or surgical in nature, and these are sometimes combined. Decisions on the management choices for CSP are prescribed based on the patient’s menstrual age, β-hCG titres, the detection of the embryo’s heart pulse, the patient’s desire to procreate and the physician’s skills. Surgical treatment involves hysterectomy as a radical treatment in cases of uterine rupture and severe haemorrhage. In addition, conservative surgical measures, including dilatation and curettage (D and C), and laparotomy or laparoscopy, are used for uterine evacuation and ligation of both uterine defects and a hypogastric artery, respectively.^10–13^ Medical therapy essentially includes methotrexate, which can be administered either systemically, locally or in combination.^14,15^ Finally, a medical approach is sometimes combined with bilateral uterine artery embolization, minimizing the risk of life-threatening haemorrhage.^16^

## Acknowledgements

The authors would like to thank Mr. Mutaz Bashir Almalik (Specialist of Medical Ultrasound Imaging) and Dr. Omer Alzain (Obstetric and Gynaecology Registrar) at Alnohood Teaching Hospital for their cooperation and support during writing this manuscript. English-language editing of this manuscript was provided by Journal Prep.

## Learning points

1. Intramural pregnancy with implantation in a previous caesarean section scar is probably the rarest location for an ectopic pregnancy.
2. Decisions on the management choices for caesarean scar pregnancy are prescribed based on the patient’s menstrual age, β-hCG titers, the detection of the embryo’s heart pulse, the desire to procreate and the physician’s skills.
3. In patients with suspected caesarean scar pregnancy and specific clinical conditions, as well as TVS and colour Doppler imaging findings, play a leading role in establishing a rapid and accurate diagnosis.

## Consent

Written informed consent for the case to be published (including images, case history and data) was obtained from the patient(s) for publication of this case report, including accompanying images.
